# Effects of Weak Surface Modification on Co/SiO_2_ Catalyst for Fischer-Tropsch Reaction

**DOI:** 10.1371/journal.pone.0124228

**Published:** 2015-05-04

**Authors:** Wensheng Ning, Hehong Shen, Yangfu Jin, Xiazhen Yang

**Affiliations:** 1 College of Chemical Engineering, Zhejiang University of Technology, Hangzhou, China; 2 College of Materials Science and Technology, Zhejiang University of Technology, Hangzhou, China; Queen's University Belfast, UNITED KINGDOM

## Abstract

A weak surface modification is applied to Co/SiO_2_ catalyst by hydrothermal treatment at 180°C for 5 h. Aluminum is introduced to Co/SiO_2_ catalysts during the surface modification. The effects of surface modification on Co/SiO_2_ catalyst are studied by changing the operating sequences of surface modification and cobalt impregnation in the catalyst preparation. Surface modification before cobalt impregnation makes Co_3_O_4_ particle small and dispersed into the deep part of enlarged pore in SiO_2_, while surface modification after cobalt impregnation does not obviously change the particle size of Co_3_O_4_. The improved amplitude of catalytic activity is similar for the two kinds of catalysts, but they are benefited from different factors. The content of iso-hydrocarbons in the products is increased by the surface modifications.

## Introduction

Fischer-Tropsch (FT) reaction is a key technology to convert coal, natural gas and biomass into fuels and chemicals via syngas (mixture of CO and H_2_) [1,2]. Therefore, FT reaction can eliminate the worry about liquid-fuel shortage originated from crude oil exhaustion. Fe and Co are commercial catalysts for FT reaction. Because Co catalyst has higher resistance towards re-oxidation by the produced water [3] and higher carbon efficiency for CO into hydrocarbons [4,5] than Fe catalyst, Co catalyst is optimal for FT reaction based on natural gas [4].

Due to the obtained products are high-molecular weight waxes, further process is needed to make the waxes into liquid fuels which can be used by internal-combustion engine. Some researchers tried to adjust Co catalyst for one-step synthesis of gasoline directly from syngas by coating the catalyst with zeolite membrane [6–8]. A compact zeolite membrane was formed at the outside of Co/SiO_2_ pellets after a crystallization more than 24 h, but the zeolite membrane resulted in decreased CO conversion and increased CH_4_ selectivity [6,7]. The changes of CO conversion and CH_4_ selectivity weaken the overall efficiency of FT reaction based on natural gas. With the duration time for coating zeolite on Co/SiO_2_ shortened to 12 h, CH_4_ selectivity was decreased [8]. The above results suggest that the performance of Co/SiO_2_ catalysts can be improved further by shortening the duration time of surface modification.

Considering it is lacking the knowledge about Co/SiO_2_ catalysts modified by short zeolite crystallization, we studied weak surface modification on Co/SiO_2_ catalyst by hydrothermal treatment at 180°C only for 5 h. The changes of catalyst structure and reactive performance are discussed here.

## Materials and Methods

### Catalyst preparation

**Fig 1:**
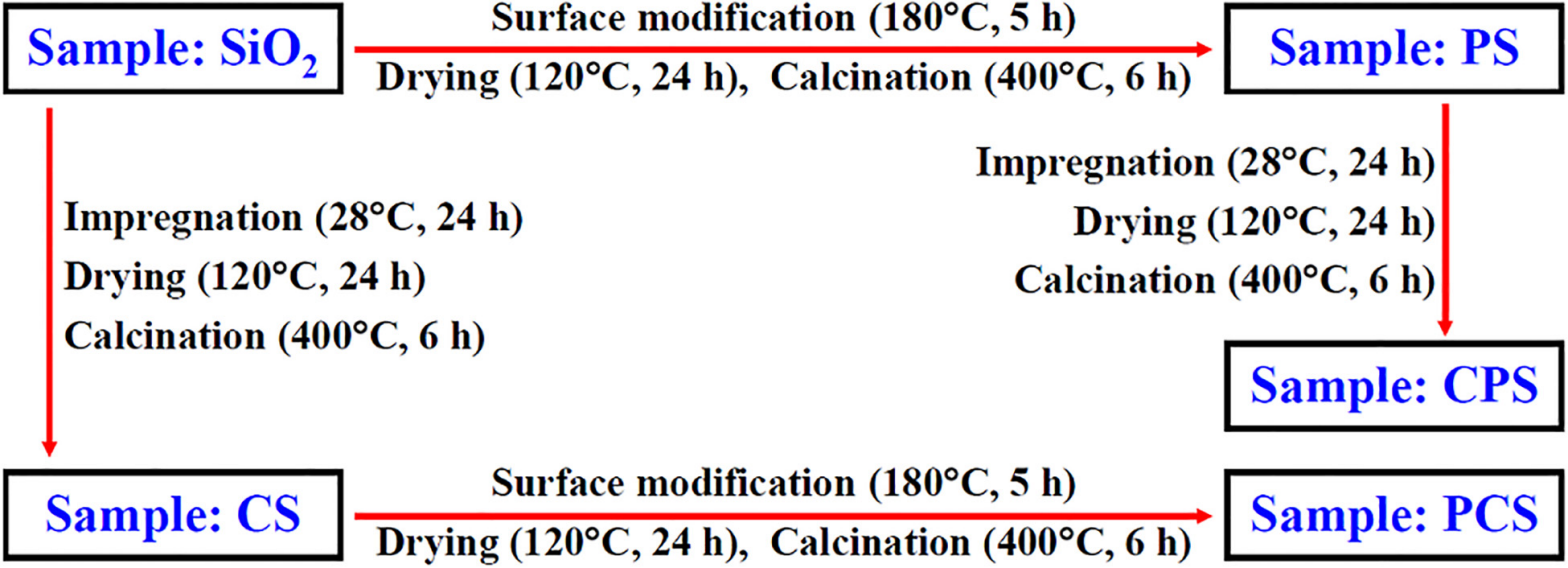
Program for catalyst preparation.

Fig 1 describes the details about catalyst preparation. Supported Co/SiO_2_ catalyst was prepared by incipient wetness impregnation of SiO_2_ (Qingdao Haiyang Chemical Co., Ltd) or modified SiO_2_ of 150–280 μm with aqueous solution of cobalt nitrate hexahydrate (Co(NO_3_)_2_·6H_2_O, AR, Sinopharm Chemical Reagent Co., Ltd). The mass ratio of Co:SiO_2_ or modified SiO_2_ was 1:10. Surface modification to SiO_2_ and Co/SiO_2_ was done in a 100 ml Teflon autoclave at 180°C for 5 h with the mixture of ethyl silicate (TEOS, AR, Shanghai Chemical Reagent Co.), tetrabutylammonlum hydroxide (TBAOH, AR, 10% water solution, Shanghai Qiangshun Chemical Reagent Co., Ltd), distilled water, dehydrated alcohol (EtOH, AR, Anhui Ante Food Co., Ltd) and aluminum nitrate nonahydrate (Al(NO_3_)_3_·9H_2_O, AR, Shanghai Zhenxin Chemical Reagent Co.). The sequence to input these raw materials into the autoclave was H_2_O, TBAOH, EtOH, TEOS, Al(NO_3_)_3_·9H_2_O and SiO_2_ (or Co/SiO_2_) and the mass ratios of them equated to 67.5: 0.26: 4.22: 1.05: 0.105: 10.

### Activity test and product analysis

**Fig 2:**
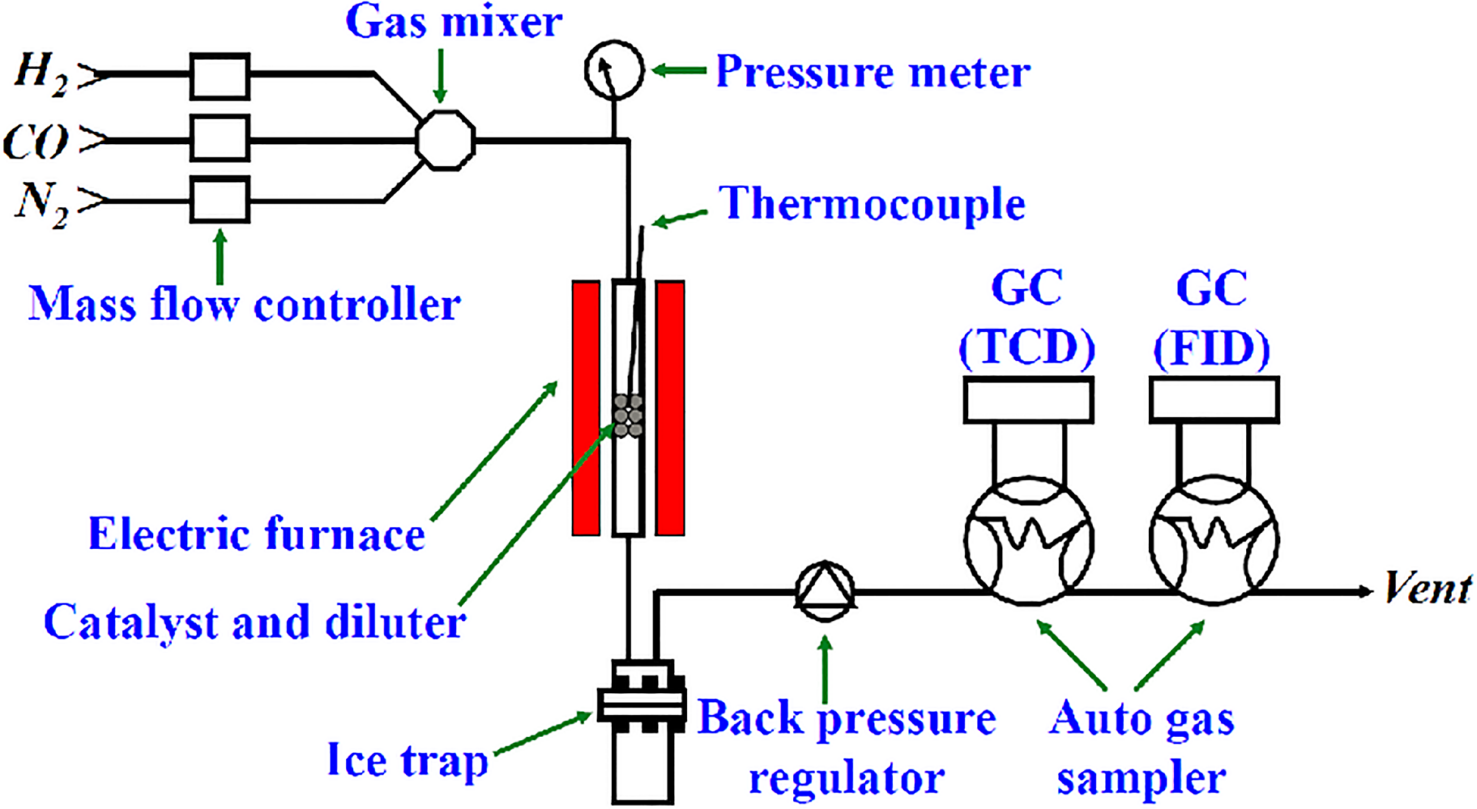
Principal scheme of the evaluation apparatus.

The reactivity of catalysts was tested in a stainless steel fixed bed reactor shown in Fig 2. A 1.0 g catalyst (150–280 μm) was mixed with 4.0 g quartz sand and they were filled into the reactor. After the catalyst was reduced in H_2_ of 3.6 L/(h·g-cat) at 400°C for 6 h, it was cooled to room temperature. Then, the feed gas was changed into reactants of 2.0 MPa. The catalyst was heated to 220°C in about 3 h for activity evaluation. The detailed testing parameters and analyzing methods are as same as those in our previous work [9].

### Characterization

Temperature-programmed reduction (TPR) was carried out in AutoChem 2910 (Micromeritics LTD.) with 5% H_2_/Ar and a TCD detector [10]. The morphology of the catalysts was observed by scanning electron microscopy (SEM, Hitachi S-4700II) which was attached with an energy dispersive spectroscopy (EDS, Thermo NORAN VANTAGE ESI). Surface area and pore structure of the samples were measured by ASAP-2020 from Micromeritics. The crystal structure of the catalysts was analyzed by X-ray diffraction (XRD, PNAlytical X’Pert Pro diffractometer) with a Cu Kα radiation source (λ = 0.15406 nm).

## Results and Discussion

### Catalyst performance in FT reaction

**Fig 3:**
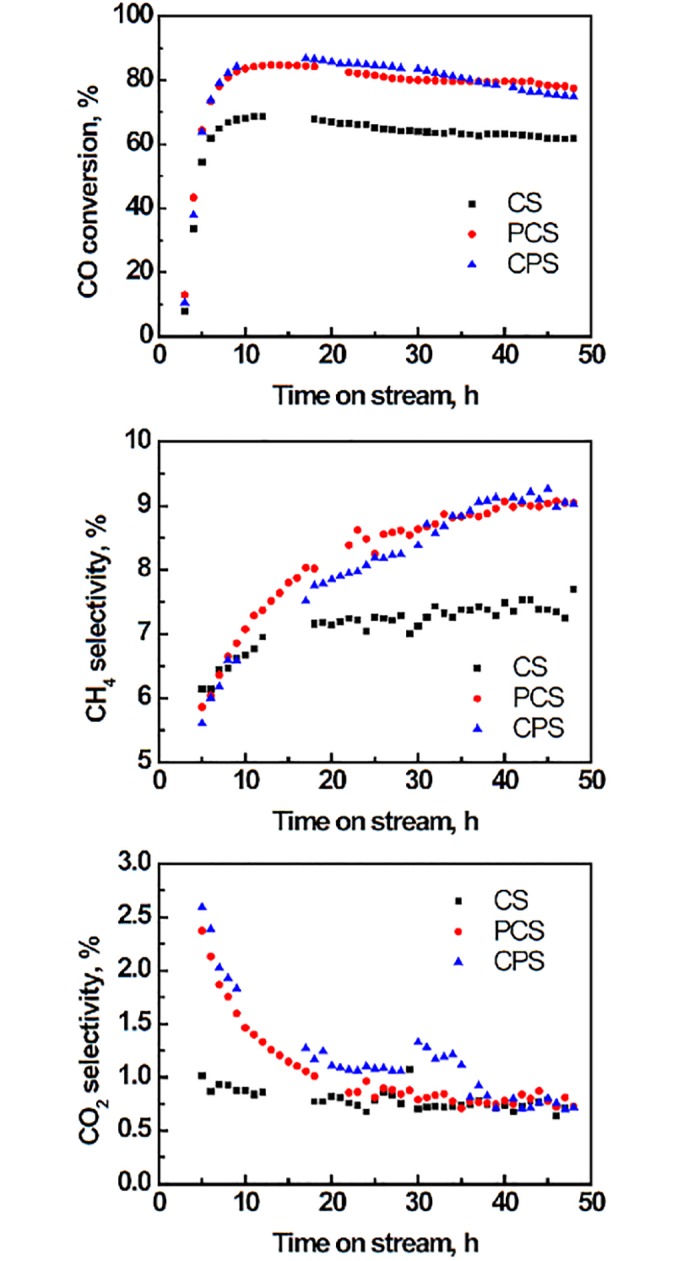
Activity and selectivity of modified Co/SiO_2_ catalysts in FT reaction. 220°C, 2.0 MPa, 1.8 L/(h·g-cat), H_2_/CO/N_2_ = 6/3/1.

Fig 3 shows the performance of catalyst CS, PCS and CPS in FT reaction. PCS and CPS have similar CO conversions which are higher than CS. Although the three catalysts show the same climbing trend for CH_4_ selectivity in the beginning, the values of PCS and CPS are finally increased about 22% based on CS. CO_2_ selectivity of CS is almost stable during the evaluation period, while that of PCS and CPS descends gradually to the level of CS. In view of CO conversion and CH_4_ selectivity, the surface structures (properties) of PCS and CPS are different from CS which was changed by the hydrothermal crystallization of 5 h. Based on the data after 40 h on stream (Fig 3), the calculation shows that the increased carbon atoms in the products of CH_4_ and CO_2_ only account for about 20% of the carbon atoms in the extra CO converted after the CS was modified. Much of the carbon from the increased CO conversion was converted into C_2_+ hydrocarbons. This improvement can be attributed to the weak surface modification of the Co/SiO_2_ catalyst for the Fischer-Tropsch reaction.

He et al. [6] coated HZSM5 membrane onto preshaped Co/SiO_2_ catalyst. The thickness of zeolite membrane is about 10 μm after hydrothermal crystallization of 2 d. The capsulated catalysts (Co/SiO_2_-zeolite) have lower CO conversion and higher CH_4_ selectivity than uncapsulated Co/SiO_2_. The thick zeolite membrane enhances long-chain hydrocarbons cracked into short-chain ones including CH_4_ [8], and increases H_2_/CO ratio around Co/SiO_2_ because H_2_ diffuses more quickly than CO through the thick membrane [6,7]. The two effects jointly raise CH_4_ selectivity of Co/SiO_2_-zeolite 44% more than Co/SiO_2_ [6]. On the contrary, about 22% rise of CH_4_ selectivity is due to the short duration of surface modification in this work. The raw materials we used are similar to those of He et al. [6] except tetrapropylammonium hydroxide solution was adopted by them, but the duration of hydrothermal crystallization was much shorter than theirs, which was probably not enough to form a consistent and thick zeolite membrane on PCS. In deed, we do not observe consistent zeolite membrane on PCS by SEM. Wang et al. studied Co/zeolite catalysts [11]. The surface area and pore size are the same for the used support NaX and NaY, but the Si/Al ratio of NaX is lower than NaY. In spite of the reduced degree of the two catalysts is similar each other, Co/NaX is more active than Co/NaY in FT reaction. There is one positive correlation between Al content and CO conversion.

Table 1 summarizes the olefin ratio and iso-hydrocarbon content in C_4_ hydrocarbons measured after 46 h on stream. The olefin ratio in C_4_ hydrocarbons is similar for the three catalysts CS, PCS and CPS, but both of the iso-olefin and iso-paraffin content in C_4_ olefins and paraffins from CS are much lower than those of PCS and CPS. The isomerization activity of Co/SiO_2_ is enhanced by the surface modification [6]. PCS has stronger isomerizing function than CPS, which is related to the sequence of surface modification in the total process to prepare the catalysts.

**Table 1:** Distribution of C_4_ hydrocarbons.

Catalyst	C_4,O_/(C_4,O_ + C_4,P_)	iC_4,O_/C_4,O_	iC_4,P_/C_4,P_
CS	0.28	0.09	0.47
PCS	0.27	0.21	0.59
CPS	0.26	0.16	0.56

O: olefin. P: paraffin. i: isomerized hydrocarbon.

In view of the duration of hydrothermal crystallization, the surface modification is weak in this work compared with those [6–8]. However, the weak surface modification is superior to the strong treatments [6–8] because it can increase the activity of Co/SiO_2_ catalysts. The degree of surface modification is worthy to be investigated further in order to balance the desired increase of iso-hydrocarbon selectivity and undesired increase of CH_4_ selectivity.

### Temperature-programmed reduction

**Fig 4:**
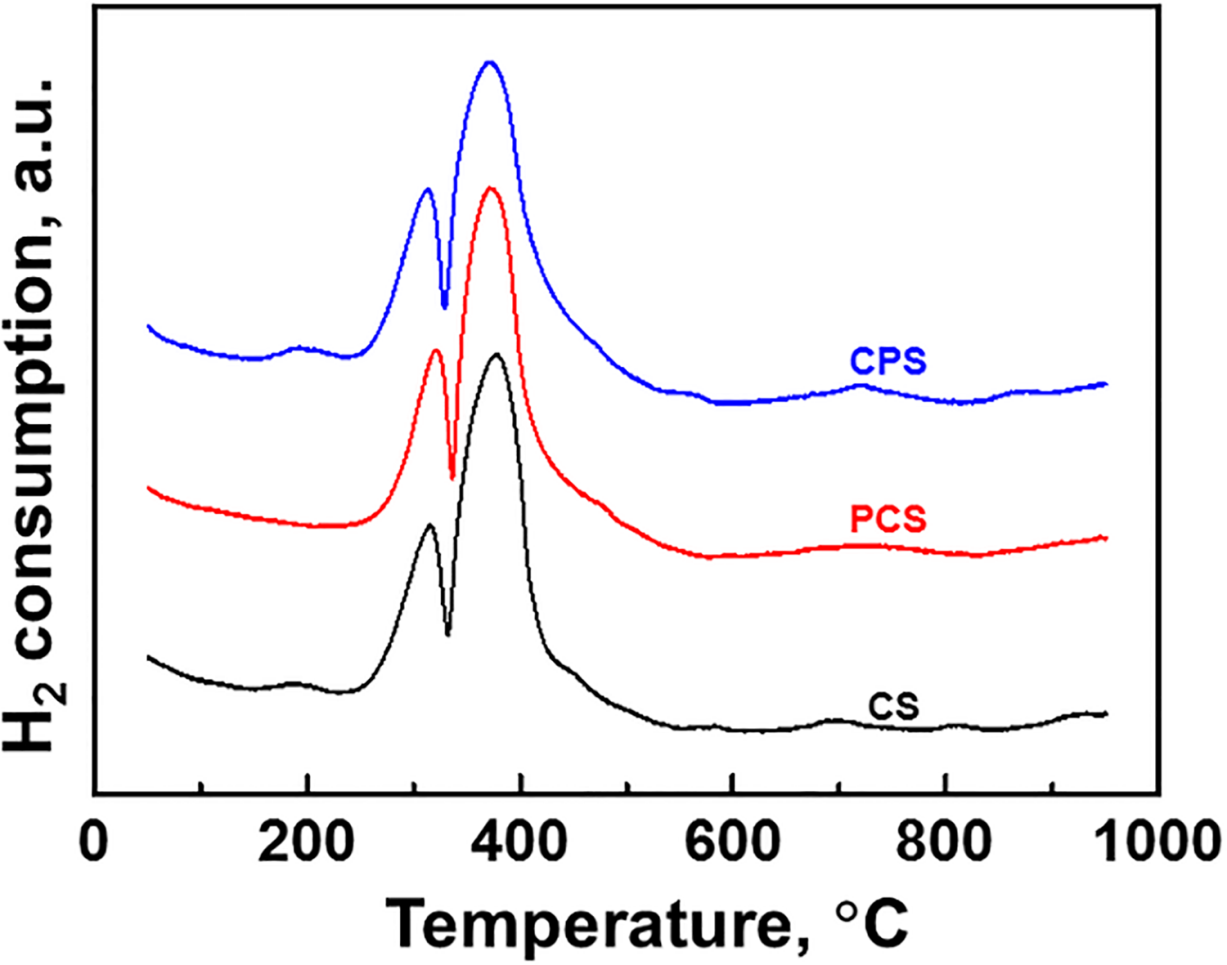
H_2_-TPR profiles of modified Co/SiO_2_ catalysts. 5% H_2_/Ar, 24L/(h·g-cat), 10°C/min.

Fig 4 compares the reducibility of CS, PCS and CPS in H_2_-TPR. There are two main peaks in the range of 250°C–520°C. They are from the two sequential reductions of Co_3_O_4_ to Co^0^ with CoO as intermediate [9,12–15]. A minor peak around 190°C appears for CS and CPS. It is resulted from the reduction of cobalt nitrate which was remained in CS and CPS in spite of they were calcinated at 400°C [9,14,15]. But the cobalt nitrate remained in CS was decomposed during the followed surface modification, because the peak around 190°C is not observed for PCS. Similarly, the peak above 650°C in PCS is weaker than those in CS and CPS. These indicate that the surface property of CS is changed by the surface modification.

### Morphology of catalysts

**Fig 5:**
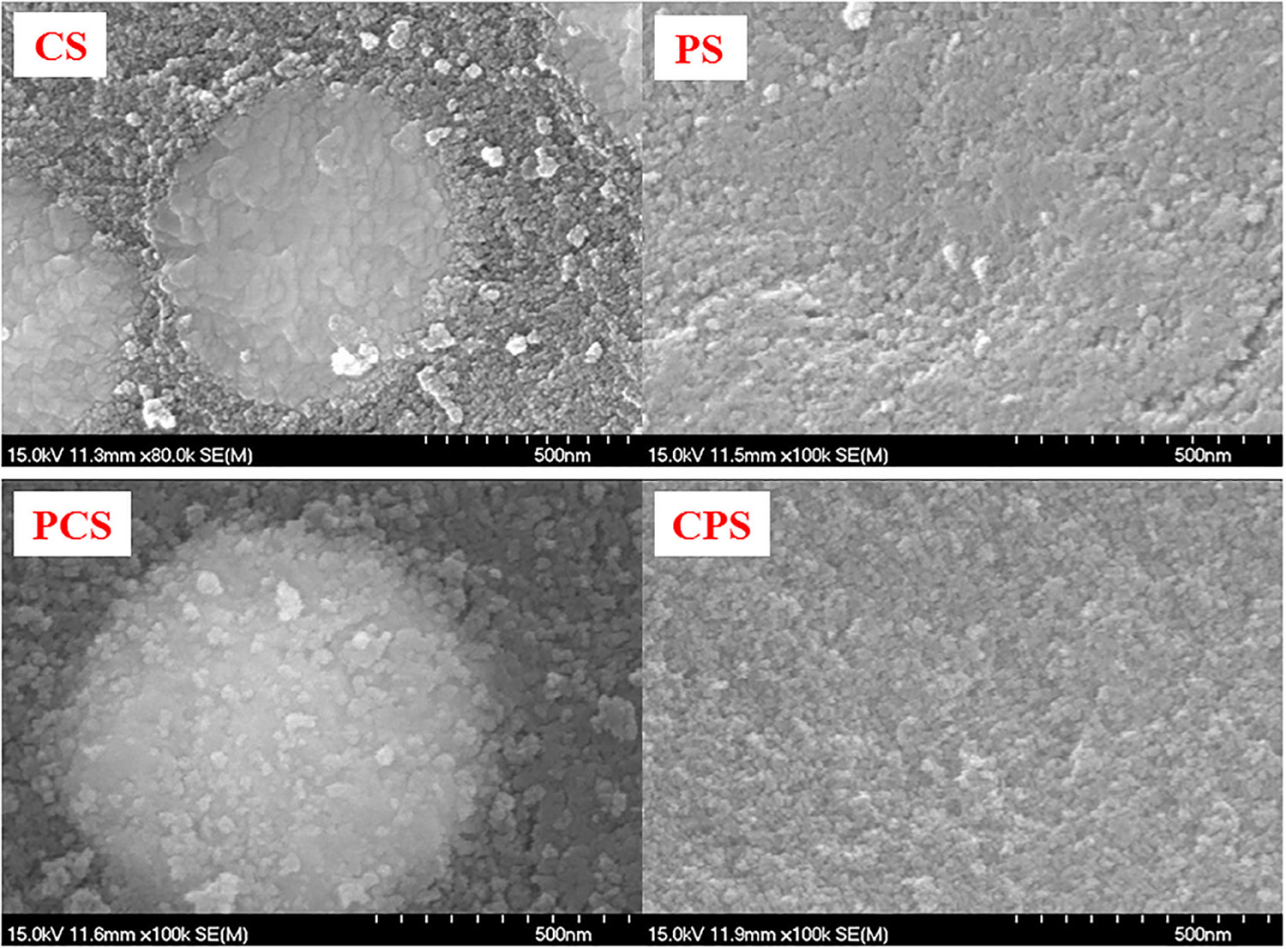
Morphology observed by SEM.

Fig 5 presents the morphology of modified SiO_2_ and Co/SiO_2_ catalysts. There is circular particles on CS and PCS with diameter about 800–850 nm. They are confirmed to be cobalt-containing species by EDS (S1 Fig). Every cobalt-containing particle on CS is agglomerated by smaller unit. The width of the small unit is less than 50 nm, which approaches to the crystal size calculated from XRD patterns. For PCS, the cobalt-containing particle is covered by plate-like additive, which is clearly seen in its right upper part. However, the modification time of 5 h is too short to form consistent zeolite membrane on PCS. On the contrary, thick and consistent zeolite membrane was formed on Co/SiO_2_ catalyst by 2 d of hydrothermal crystallization [6]. So, the surface modification is weaker in our work than those [6–8].

**Fig 6:**
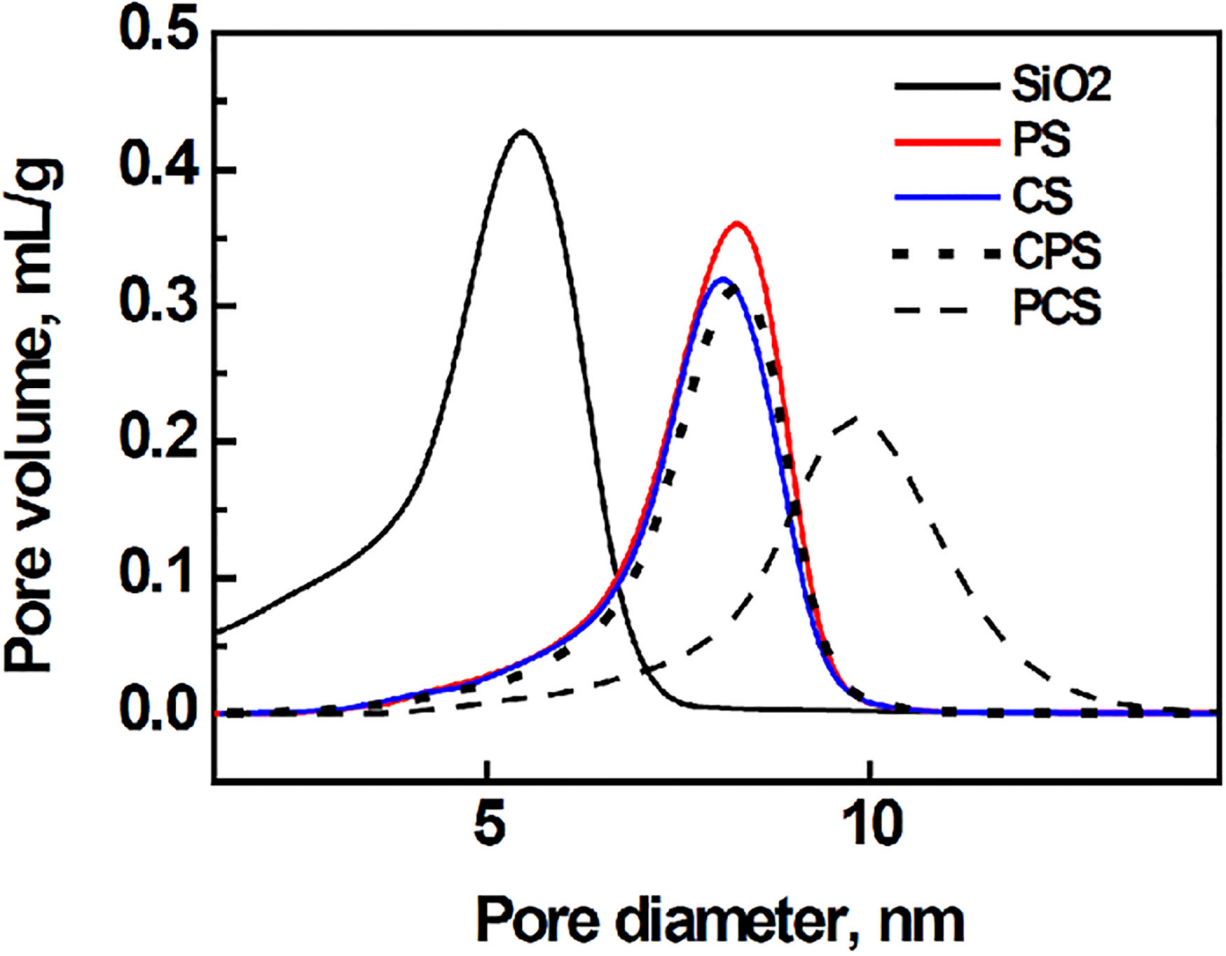
Pore size distribution.

The coarse surface of SiO_2_ becomes smooth after the surface modification as shown by PS in Fig 5. Surprisingly, there are no circular particles on CPS as those observed on CS and PCS. The surface composition of Co/SiO_2_ catalysts measured by EDS is given in Table 2. In general, the EDS result can be accepted as quasi-quantified datum. The detected Al on CS is from the impurity in SiO_2_. There is more Al on PCS and CPS than CS. It reflects that Al was introduced to PCS and CPS by surface modification [6]. The detected Co content on CPS is less than half of CS and PCS, and it is understandable because of no evident cobalt-containing particles on CPS (Fig 5). According to Table 2 and Fig 6, the pore size of SiO_2_ was enlarged after the surface modification as presented by PS. Therefore, the impregnated cobalt is easily to enter into the deep part of pores and result in its less distribution on the outer surface of CPS.

**Table 2:** Surface composition and texture of Co/SiO_2_ catalysts.

Sample	Surface composition (atom %)				BET surface area (m^2^/g)	Pore volume (mL/g) ^a^	Average pore diameter (nm) ^a^
	Si	Co	Al	O			
SiO_2_	-	-	-	-	637.7	0.84	5.47
PS	-	-	-	-	344.0	0.80	8.25
CS	27.97	5.84	0.29	65.91	302.8	0.71	7.49
PCS	28.54	4.20	0.48	66.78	243.2	0.66	10.21
CPS	26.11	2.04	0.50	71.35	296.1	0.69	8.25

^a^: These values were calculated by BHJ method from the desorption isotherm.

### Texture of catalysts

Table 2 lists the BET surface area and pore distribution of SiO_2_ and catalysts measured by low temperature N_2_ physical adsorption. Every operation, surface modification or cobalt impregnation, makes the catalyst surface area decrease. Correspondingly, the pore volume becomes small, too. Compared with SiO_2_, the other samples possess enlarged average pore diameter. The detailed change of pore structure is shown in Fig 6. There is only one peak in the pore size distribution for all the studied samples. It indicates that the enlargement of pore size is from the merge of small pores in SiO_2_ due to the collapse of pore wall occurred during or after impregnation / surface modification. It has been reported that the water used as solvent can destroy the pore structure of small diameter when intrapore water is removed by drying [16,17]. Because the pattern of CPS is almost overlapped with that of PS except the decline in the peak top, the impregnated cobalt was deposited into the deep of pore, rather than distributed on the pore mouth or the outer surface. The latter two distributions would markedly let the peak shifted left or the peak height of CPS downturn largely relative to PS, respectively.

Kang et al. reported that the surface area and average pore size were suppressed and a bimodal pore size distribution was formed after a complete crystallization of ZSM5 on Co/SiO_2_, which was assigned to the pore blockage of Co/SiO_2_ catalyst with ZSM5 particle [8]. Based on the works of Kang et al. [8] and ours, it can be inferred that the hydrothermal crystallization mainly enlarges the pore size of SiO_2_ during the initial period which is not shorter than 5 h, then zeolite is formed on SiO_2_ with extended hydrothermal duration.

Although PCS has smaller surface area and pore volume than CS, its average pore diameter is larger than CS. The large pore is beneficial for the diffusion of reactants to catalyst and products from catalyst. It is a possible reason for the increased CO conversion on PCS compared with CS.

### Crystal structure of oxidized catalysts

**Fig 7:**
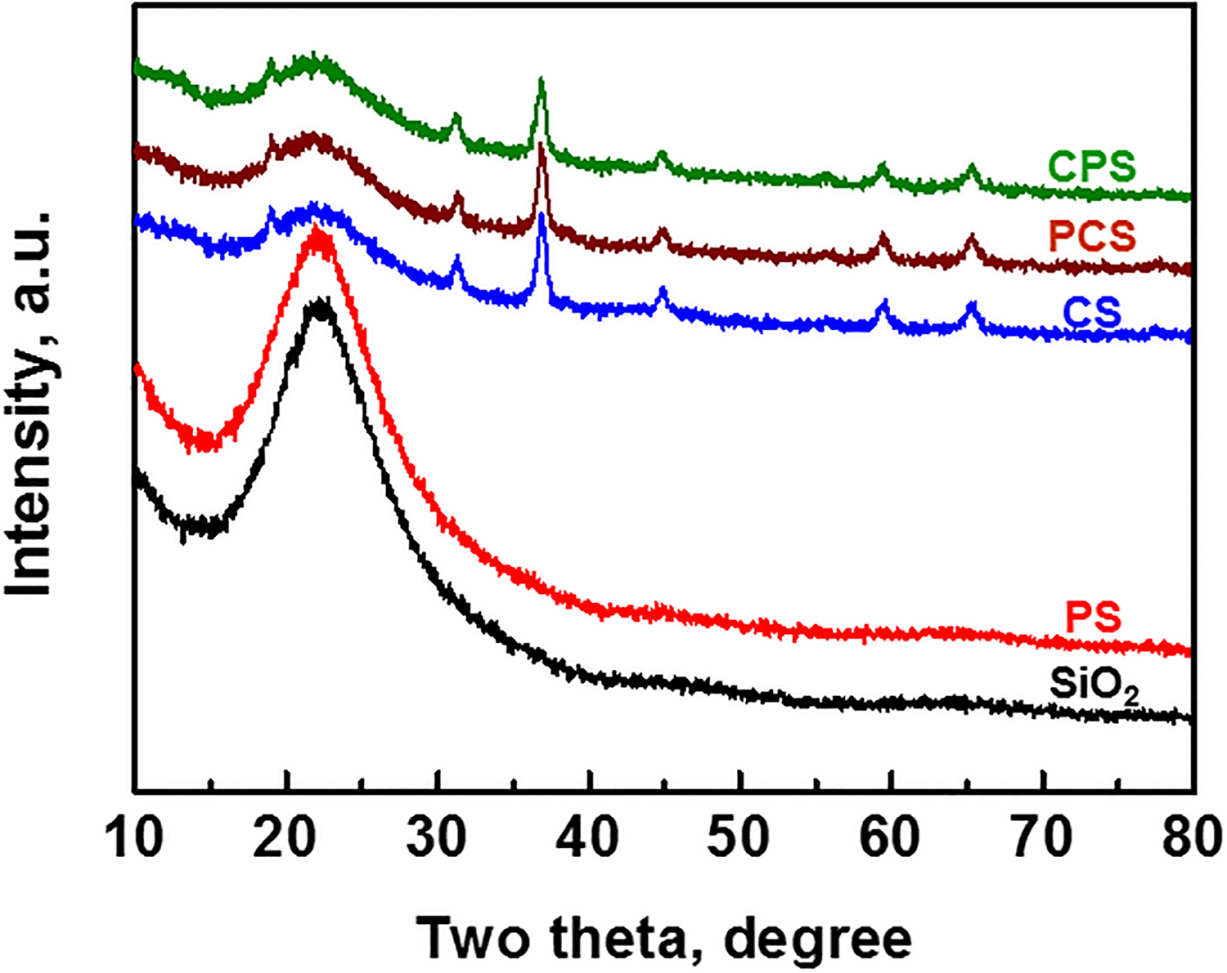
XRD patterns of oxidized catalysts.

Fig 7 is the XRD patterns of SiO_2_ and oxidized catalysts. There is a wide peak around 22.1° for SiO_2_, which remains in PS, CS, PCS and CPS in spite of the support is modified or impregnated or modified + impregnated. Besides the SiO_2_ peak in CS, PCS and CPS, other peaks are assigned to Co_3_O_4_ species (PDF: 80–1532). Calculated with Scherer equation [7,8,18,19] based on 36.8° peak, the crystal particle size of Co_3_O_4_ is 21 nm, 25 nm and 15 nm in CS, PCS and CPS, respectively. The hydrothermal condition to prepare PCS may induce the growth of Co_3_O_4_ particles by the decomposition of cobalt nitrate remained in CS. Kang et al. [8] thought the phenomenon possibly resulted from Co_3_O_4_ migration from Co/SiO_2_ to the outer surface of ZSM5. The Co_3_O_4_ size of CPS is the smallest among CS, PCS and CPS. It is a reasonable result because the cobalt-containing particle is deposited into the pore of CPS and its size is limited by the pore. The Al added on SiO_2_ by the surface modification is helpful to form small Co_3_O_4_ particles on PS [20], too. We had observed that the size of cobalt-containing particle was decreased with Al_2_O_3_ addition to ZnO [9].

Although PCS and CPS show similar increased CO conversion compared with CS (Fig 3), the underlying factors are different. The improvement to CPS is originated from small Co_3_O_4_ particle that can supply more active site after H_2_ reduction [18,21,22], while PCS benefits from Al addition [11] and eased diffusion of reactants to catalyst.

## Conclusions

Co/SiO_2_ catalysts are modified in a hydrothermal environment introducing Al to the catalysts. The sequence of surface modification in the total process to prepare Co/SiO_2_ catalysts has different influences on catalysts’ structure and performance. PCS, with surface modification after cobalt impregnation, possesses similar particle size of Co_3_O_4_ to CS, but it is more active than CS to convert CO in FT reaction. The improvements are benefited from Al addition and enhanced diffusions of reactants to catalyst and products from catalyst. CPS, surface modification before cobalt impregnation, has smaller Co_3_O_4_ size than CS which would raise the active sites and increase its catalytic activity. The surface modification, irrespective of its sequence, increases the isomerization activity of Co/SiO_2_ in FT reaction. The current work verifies that a weak surface modification can combine the two functions of hydrocarbon synthesis and isomerization into Co/SiO_2_ catalysts. The modified degree is worthy to be investigated further to balance the desired increase of iso-hydrocarbon selectivity and the undesired increase of CH_4_ selectivity.

## Supporting Information

S1 Fig The surface distribution of element Co, Si, Al and O on catalyst CS, PCS and CPS measured by EDS. (PDF)
