# Supplementary figures and images for: Effects of Weak Surface Modification on Co/SiO2 Catalyst for Fischer-Tropsch Reaction
Source: PLoS One. 2015 May 4;10(5):e0124228. doi: 10.1371/journal.pone.0124228 (PMC4418669; doi:10.1371/journal.pone.0124228)

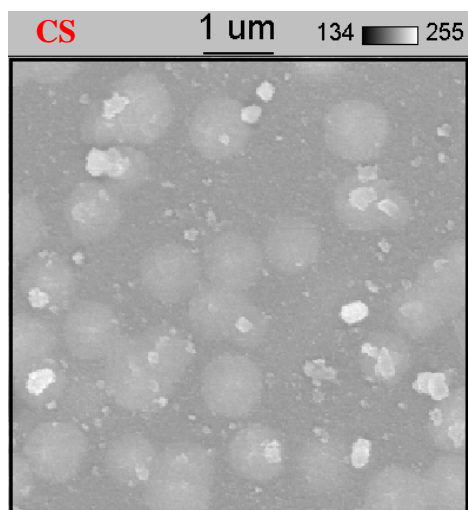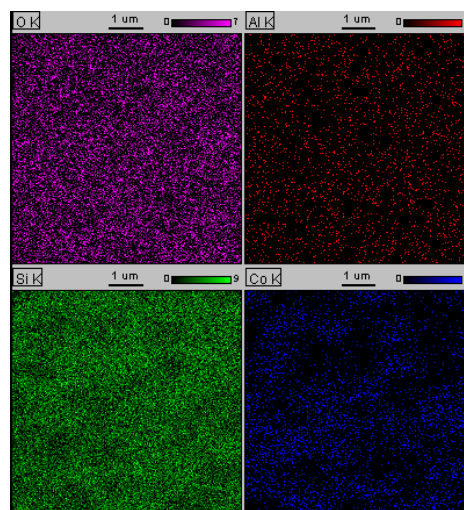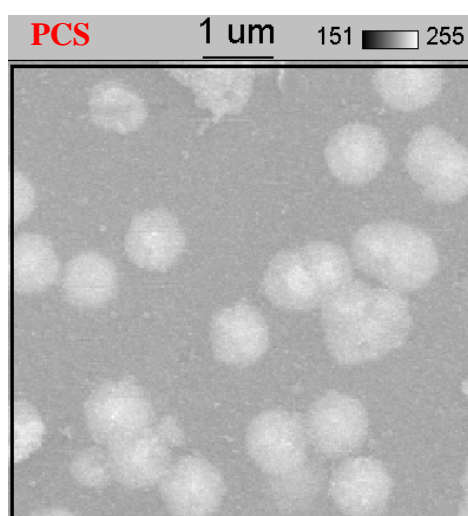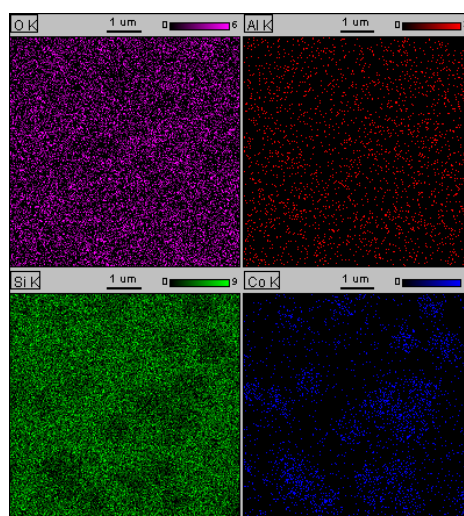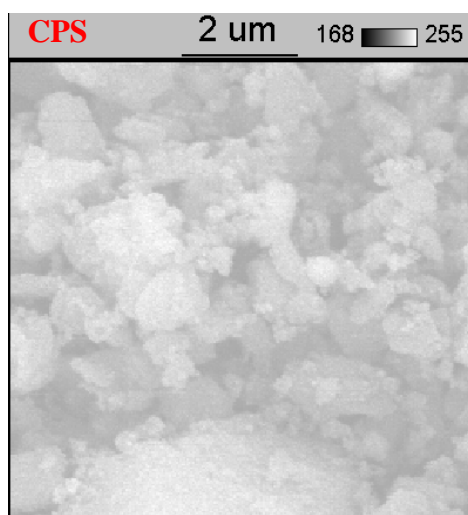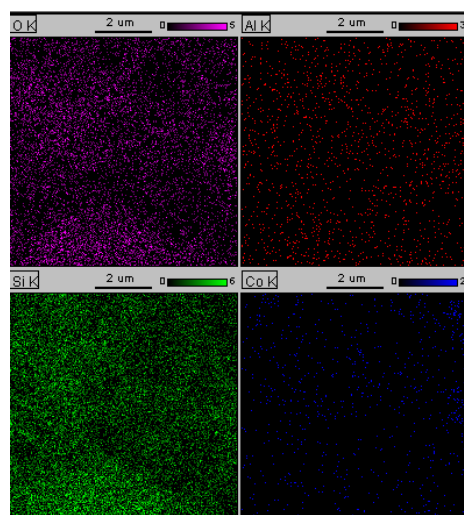

Supplement: S1 Fig — (PDF) [file pone.0124228.s001.pdf]
